# CMOS MEMS Fabrication Technologies and Devices

**DOI:** 10.3390/mi7010014

**Published:** 2016-01-21

**Authors:** Hongwei Qu

**Affiliations:** Department of Electrical and Computer Engineering, Oakland University, 2200 N. Squirrel Road, Rochester, MI 48309, USA; qu2@oakland.edu; Tel.: +1-248-370-2205

**Keywords:** CMOS (complementary metal-oxide-semiconductor), MEMS (micro-electro-mechanical systems), CMOS MEMS, integration, sensors, actuators

## Abstract

This paper reviews CMOS (complementary metal-oxide-semiconductor) MEMS (micro-electro-mechanical systems) fabrication technologies and enabled micro devices of various sensors and actuators. The technologies are classified based on the sequence of the fabrication of CMOS circuitry and MEMS elements, while SOI (silicon-on-insulator) CMOS MEMS are introduced separately. Introduction of associated devices follows the description of the respective CMOS MEMS technologies. Due to the vast array of CMOS MEMS devices, this review focuses only on the most typical MEMS sensors and actuators including pressure sensors, inertial sensors, frequency reference devices and actuators utilizing different physics effects and the fabrication processes introduced. Moreover, the incorporation of MEMS and CMOS is limited to monolithic integration, meaning wafer-bonding-based stacking and other integration approaches, despite their advantages, are excluded from the discussion. Both competitive industrial products and state-of-the-art research results on CMOS MEMS are covered.

## 1. Introduction

Last decade has seen the rapid maturity of the MEMS (micro-electro-mechanical systems) industry. MEMS are now prevalent in our daily life. Probably the most popular gadget in which MEMS have significant applications, known or unknown to the user, is a smart phone. MEMS motion sensors, including accelerometers and gyroscopes with 6 degrees of freedom, along with MEMS microphones, pressure sensors, magnetometers, *etc.*, have greatly contributed to the operation of the smart phone, which can be considered a little do-everything device. In automobiles, in addition to the well-known MEMS devices for vehicle performance and safety, such as pressure sensors for manifold intake vacuum measurement, smart tire pressure monitoring, accelerometers for airbag deployment, accelerometers and gyroscopes for electronic stability programs (ESP), inclinometer for sliding prevention, *etc.*, MEMS have found new applications in environmental monitoring for driving and riding comfort and safety improvements. In other areas, MEMS are serving us in inkjet printers with MEMS print heads and portable electronics with MEMS resonators as frequency references, just to name a few. In the past few years, MEMS market has enjoyed consecutive double-digit growth. The worldwide MEMS market is predicted to top 22 billion U.S. dollars by 2018 [1].

The ultimate goals for MEMS have been and will continue to be continuous miniaturization, expanded functionalities, lower cost, and improved performance and reliability. The purpose of MEMS demands direct integration of mechanical structures with electronics that are normally fabricated by CMOS (complementary metal-oxide-semiconductor) technologies. In the last couple of decades, with breakthroughs in individual technologies and enabling tools, great efforts have been made to integrate MEMS structures with integrated circuits (IC) on a single CMOS substrate, for the so-called monolithic CMOS MEMS integration. Integration of those subsystems into new materials such as silicon-on-insulators (SOI) has been attempted as well. Numerous microfabrication and integration approaches have been attempted [2].

CMOS MEMS are micromachined systems in which MEMS devices are integrated with CMOS circuitry on a single chip to enable miniaturization and performance improvement. CMOS MEMS also refers to microfabrication technologies and processes that are involved in the creation of these integrated devices. One of the best-known commercial monolithic CMOS-MEMS devices is the digital micromirror device (DMD) manufactured by Texas Instruments [3]. In the research community, one of the pioneering efforts for CMOS MEMS transducers was made by H. Baltes and his coworkers at the Swiss Federal Institute of Technology, Zurich (ETH) [4]. They employed both wet bulk silicon micromachining and surface micromachining techniques in the fabrication of integrated CMOS MEMS devices. With advancement of both CMOS and micromachining technologies, CMOS MEMS have also evolved tremendously in recent years [5].

From a historical perspective, this paper summarizes a variety of CMOS MEMS monolithic integration technologies and associated devices that have made use of the respective technologies. Due to the huge diversity of CMOS-MEMS integrated devices and systems, though other systems and associated technologies such as CMOS-bioMEMS devices and integration for fluid handling and analysis are emerging, as reported in references [6,7,8,9,10] and thereafter, only conventional MEMS devices, including a variety of physical sensors, resonators, and actuators, are used as examplesl featuring the respective CMOS MEMS technologies.

## 2. Classification of CMOS MEMS Technologies

MEMS can be integrated with CMOS electronics monolithically in number of different ways. Despite the availability of various materials used in CMOS MEMS, one common way to categorize CMOS MEMS technologies is from the perspective of manufacturing processes. Based on the sequence of processing the electronic circuitry and MEMS structures, CMOS-MEMS technologies can be classified into three categories: pre-CMOS, intra-CMOS and post-CMOS, in which the formation of MEMS is prior to, intermediate with or after the fabrication of CMOS or BiCMOS circuitries [11]. Due to its greater flexibility of accessibility to manufacturing foundries, post-CMOS approaches are more widely utilized than the others. The following sections depict these CMOS MEMS integration technologies with exemplary devices. Focus has been placed on post-CMOS MEMS integrations.

## 3. Pre-CMOS MEMS

**Figure 1:**
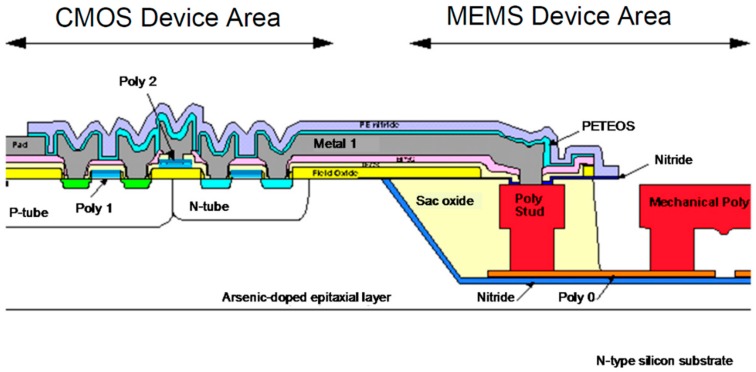
Cross-section of CMOS (complementary metal-oxide-semiconductor) and MEMS micro-electro-mechanical systems) in the recess trench in pre-CMOS integration [12].

It is widely accepted that pre-CMOS technologies are represented by the modular integration process originally developed at Sandia National Laboratories (SNL), called the integrated MEMS (iMEMS) process. As suggested by the name, in pre-CMOS technology, MEMS structures are pre-defined and embedded in a recess trench in a silicon wafer and the recess is then filled with oxide or other dielectrics, as illustrated in the cross-section in Figure 1. The wafer is then planarized prior to the following process steps for CMOS electronics [12]. In this “MEMS first” process, although MEMS structures are pre-defined, a wet etch after the completion of the standard CMOS processes is required to release the pre-defined MEMS structures. Due to the involvement of a photolithography process needed for patterning the MEMS in the recess, the thickness of the MEMS structures is constrained by the lithographical limit related to achievable focus depth.

**Figure 2:**
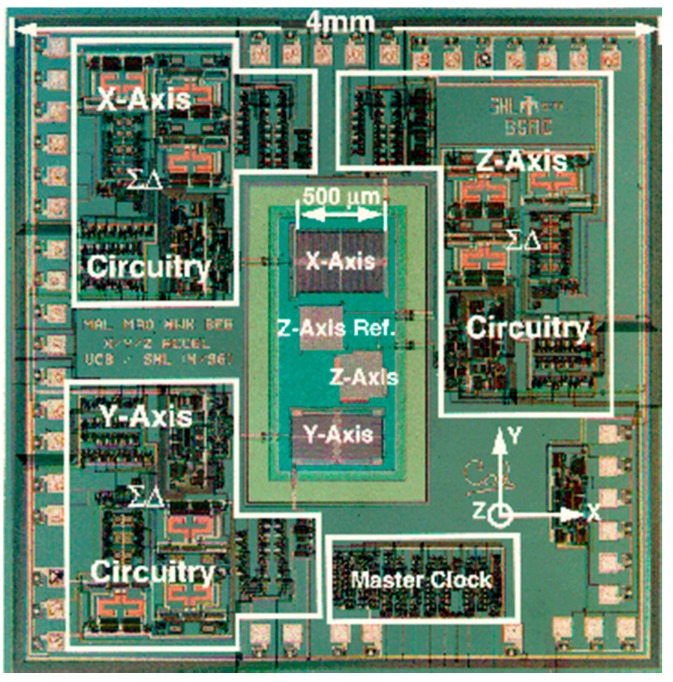
Die photo of a 3-axis accelerometer implemented by iMEMS processes [13].

Figure 2 shows a die photo of an integrated 3-axis accelerometer that was developed by Lemkin *et al.* at the Berkeley Sensors and Actuator Center (BSAC) and fabricated using the iMEMS process at Sandia [13].

Other methods for the formation of MEMS structures in pre-CMOS technologies, including wafer bonding and thinning for epitaxial and SOI wafers in which MEMS are pre-fabricated, have also been reported in the fabrication of a variety of MEMS devices. Since this paper focuses on monolithic integration of CMOS and MEMS, only SOI CMOS MEMS will be introduced in Section 5.3.

## 4. Inter-CMOS MEMS

**Figure 3:**
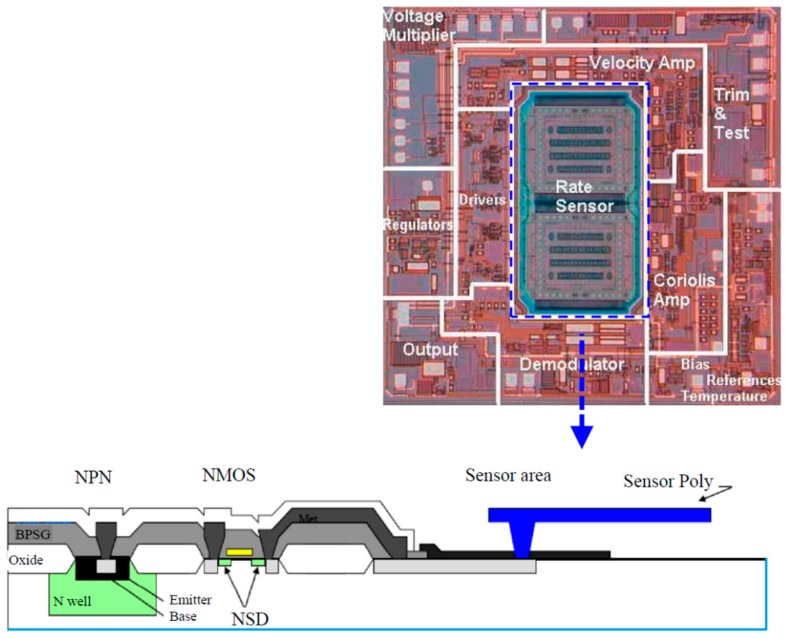
Die photo of an integrated gyroscope by Analog Devices [15].

With a dedicated facility, in the early 1990s, Analog Devices, Inc. (ADI), Norwood, MA, USA, specifically developed a MEMS technology based on its BiCMOS process. This technology, trademarked as “iMEMS” and originally dedicated to manufacturing CMOS-MEMS accelerometers and gyroscopes, is an intermediate-CMOS MEMS, or inter-CMOS MEMS, technology in which CMOS process steps are intertwined with additional polysilicon thin film deposition and micromachining steps to form sensor structures [14]. Figure 3 shows a die photo of an integrated gyroscope (ADXRS series) from Analog Devices Inc. [15]. In wafer fabrication, sensor polysilicon thin films deposition and release are interleaved by contact formation and metallization in the surrounding CMOS circuits [16]. ADI has also extended this inter-CMOS fabrication to its SOI CMOS MEMS products such as high-*g* accelerometers [17]. Infineon, another major automobile sensor provider, also manufactures its integrated capacitive pressure sensors using the inter-CMOS approach. The perforated polysilicon electrode membrane is released by wet etching of SiO_2_ prior to completion of CMOS circuity [18]. This series of integrated sensors is still in volume production and they are widely used in smart tire pressure monitoring systems (STPM) and manifold absolute pressure measurement in automobiles [19].

## 5. Post-CMOS MEMS

It is obvious from the introduction of pre- and inter-CMOS MEMS that there are some critical limitations for these two technologies. In addition to the dimension limits of the polysilicon sensor structures and the thermal processing of the polysilicon that may affect the CMOS portion of the device, the major concern for the integration is the need for dedicated manufacturing foundries and the higher cost associated with it. With the standardization of the CMOS fabrication process and availability of various foundry services in the past decade, post-CMOS MEMS integration with flexible manufacturing accessibility and cost effectiveness has proven to be an attractive option, especially for the research community.

In contrast to the previous two, in post-CMOS MEMS, all MEMS process steps are performed after full completion of the CMOS fabrication. Post-CMOS integration can date back to the late 1970s when the first generation of integrated MEMS devices—silicon pressure sensors—were demonstrated [20]. In fabrication of single crystal silicon diaphragms for pressure sensing using wet etching, the completed circuit elements on the front side of the diaphragm are normally protected by SiO_2_ or, in many cases, by acid-resistant polymers. The advantages of post-CMOS MEMS over pre- and inter-CMOS MEMS include flexibility in foundry selection, and low cost due to the independent processing of the CMOS and MEMS portions of the integrated devices. The flexibility of foundry access makes it possible to take full advantage of both advanced CMOS circuit technologies and optimal MEMS structures based on the available technologies used by the foundry. This is particularly attractive to the research community in exploration of state-of-the-art for MEMS. However, in implementation of post-CMOS MEMS, some CMOS design rules may need to be changed to accommodate MEMS structure design in the CMOS design stage. Meanwhile, post-CMOS microfabrication should be carefully designed, particularly considering the thermal budget, so as not to affect the on-chip CMOS electronics.

According to how MEMS structures are formed relative to CMOS circuitry, post-CMOS MEMS technologies fall into two categories: additive and subtractive to the CMOS. In additive post-CMOS MEMS, structural materials are deposited on the top of CMOS substrate, whereas in subtractive post-CMOS MEMS, MEMS structures are created by selectively etching CMOS layers including the substrate if necessary. Apparently, additive post-CMOS MEMS methods require more stringent material compatibility with the CMOS technologies used. Thus they are less utilized than subtractive post-CMOS MEMS.

### 5.1. Additive MEMS Structures on CMOS Substrate

**Figure 4:**
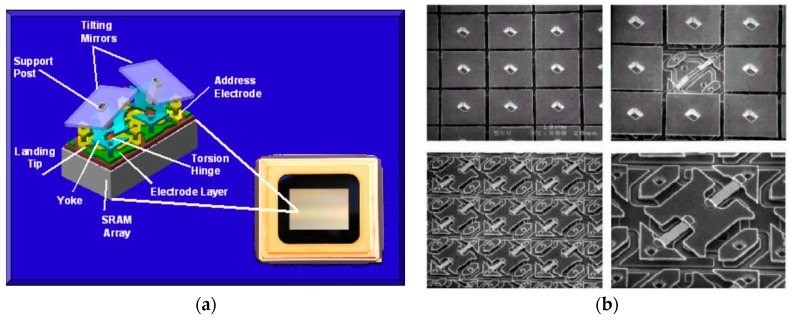
Schematic of two digital mirror device (DMD) mirror-pixels in a digital light processing (DLP) cinema chip (**a**) and scanning electron microscope (SEM) image of an array of DMD micromirrors (**b**). From www.dlp.com.

In additive post-CMOS MEMS, metals, dielectrics or polymers are deposited and patterned to form MEMS structures normally on top of the CMOS layers. Some commercial MEMS products are fabricated using additive post-CMOS MEMS approaches. In this category, the best known product is probably the digital mirror device (DMD), the core of the digital light processing (DLP) technology developed by Texas Instruments. In a DMD, tilting mirror plates and their driving electrodes are fabricated directly on top of CMOS circuits. Three sputtered aluminum layers are used to form the top mirror plate and the two parallel-plate electrodes for electrostatic actuation, respectively. The driving electrodes are addressed via a CMOS memory cell. To release the mirror plate and top electrodes in the post-CMOS MEMS fabrication of the mirrors, deep-UV hardened photoresist is used as the sacrificial layer. Figure 4 depicts two DMD pixels in a DLP cinema chip.

Other materials have also been used in attempting to create MEMS structures, especially frequency management devices, on top of CMOS layers. A good review of related devices has been conducted by Uranga *et al.* [21]. Some representative devices are further introduced as follows. A monolithic integrated nickel resonator was demonstrated by UC Berkeley, in which low-temperature electrochemically plated nickel serves as structural material for large resonator arrays and SiO_2_ as a sacrificial layer in reactive ion etching (RIE) release of the device [22]. Sandia National Laboratory (SNL) has recently demonstrated a process for post-CMOS integration of aluminum nitride (AlN) atop CMOS substrates. The structural material of AlN is deposited at 350 °C, and therefore the integration is compatible with the standard CMOS process [23]. As a demonstration of the integration, an AlN high-frequency filter with integrated transduction elements and a resonant accelerometer have been fabricated. The demonstrated resonators have an operating frequency range of 500 kHz to approximately 1 GHz, and a multiple-frequency tuning scheme can be implemented. In contrast to most capacitive MEMS resonators, the demonstrated resonators are based on the piezoelectric effect of AlN thanks to its large quality factor. The reported resonant accelerometer achieves a noise floor of 565 mg/√Hz for accelerations from 275 to 1100 Hz. Other AlN devices have also been attempted using the above technology, including tunable resonators with various bandwidth [24,25]. In all the devices, structural AlN is sputtered at 350 °C, which is CMOS-compatible. Other metals used in the devices, including metals such as tungsten, Ti/TiN/Al and insulator SiO_2_, are post-CMOS compatible and can be deposited and etched using standard CMOS tools. The AlN structural release is performed by sequential dry etches of SiO_2_, AlN and isotropic Si undercut in SF_6_ or XeF_2_. Despite its demonstrated feasibility, targeted wafer level integration of AlN to SNL’s 0.35 μm, 3.3 V SOI CMOS process is still under way. Similar to the above approach, in another effort by Uranga *et al.* at Autonomous University of Barcelona, a nanometer scale bimetallic nitride resonator capable of dual-clock tuning has been fabricated using a commercial Silterra 0.18 μm technology [26]. In this conception demonstration, in addition to the CMOS compatible structural implementation that features a small gap for enhanced coupling, a metal cap for hermetic vacuum packaging of the device is also produced.

Another representative of additive post-CMOS MEMS technology is the polycrystalline SiGe/Ge CMOS-MEMS technology from the Berkeley Sensors and Actuator Center. In this technology, polycrystalline SiGe deposited on CMOS is used as an MEMS material. Germanium or SiO_2_ thin film are used as sacrificial materials. The deposition and annealing temperatures for SiGe and Ge are below 475 °C, which is safe for the CMOS metallization. Various integrate MEMS devices, including inertial sensors, resonators and data storage devices, have been fabricated using this technology [27,28,29]. This technology has been commercialized by Silicon Clock, which was purchased by Silicon Laboratory in 2010, for manufacturing integrated MEMS oscillators [30].

Although not largely adopted later on, polysilicon was also attempted as an MEMS material for additive post-CMOS integration. However, when polysilicon was used as the structural material on top of CMOS, also introduced by BSAC in the early 1990s, the aluminum interconnection in standard CMOS technology must be replaced with refractory metals such as tungsten, to survive in the high temperature treatment for polysilicon thin film. An integrated accelerometer was demonstrated using this technology [31]. In some other circumstances where CMOS protection is well designed, electroplating can also be used to grow microstructures on top of CMOS electronics. In reference [32], accelerometer arrays with large proof mass and post-CMOS fabricated using electroplated gold are described. Copper passive components have also been demonstrated in post-CMOS fabrication of RF MEMS structures [33].

Some representative additive post-CMOS MEMS processes, along with respective materials used, are summarized in Table 1.

**Table 1:** Representative additive post-CMOS MEMS technologies and devices.

Authors and References	Institute	Structural Material	Sacrificial Material	Interconnect Material	Year
Hornbeck [34]	Texas Instruments	Al	Photoresist	Al	1989 (invented in 1987)
Yun *et al.* [31]	UC-Berkeley	Polysilicon	SiO_2_	W/TiN	1992
Franke *et al.* [35]	UC-Berkeley	Poly-SiGe	Ge or SiO_2_	Al	1999
Huang *et al.* [22]	UC-Berkeley, University of Michigan	Nickel	SiO_2_	TiN	2008
Wojciechowski *et al.* [25]	Sandia National Laboratory	AlN	Si	W/Ti/TiN	2009
Uranga *et al.* [26]	Autonomous University of Barcelona	Bimetallic Nitride	Polymer	W	2015
Sedky *et al.* [36]	IMEC	Poly-SiGe	Ge	Al	1998
Yamane *et al.* [32]	Tokyo Institute of Technology	Gold	Polyimide	Al	2013
Li *et al.* [33]	Shanghai Institute of Microsystems	Copper	Photoresist	Al	2008
Severi *et al.* [29]	IMEC, Intel	Poly-SiGe	SiO_2_	Doped SiGe	2010

### 5.2. Subtractive Post-CMOS MEMS

In these devices and technologies, back end of line (BEOL) CMOS thin film stacks including interconnected metal layers and vias, polysilicon layers, and insulating SiO_2_, are made use of in forming MEMS structures. Alternatively, silicon substrate can also be used as part of MEMS structures. These materials are patterned and removed partially or completely by wet or dry etching methods to form the MEMS structures. This section describes the thin-film and bulk CMOS MEMS formed by such subtractive processes.

#### 5.2.1. Subtractive CMOS MEMS by Wet Etching

The first generation of CMOS MEMS sensors was fabricated using a post-CMOS subtractive process in which silicon substrate was completely or partially removed using a wet etching method, leaving behind thin-film or bulk MEMS structures [4]. For thermal sensors in which beams or membranes consist of dielectric layers, the substrate silicon is normally etched away completely to obtain thermally isolated structures. For this case, the silicon dioxide membrane can act as an intrinsic etch stop layer in backside silicon anisotropic wet etching using KOH, ethylene diamine-pyrocatechol (EDP) or tetramethylammonium hydroxide (TMAH). A medical tactile sensor array was also reported in which the aluminum sacrificial layer was etched from the backside of the wafer after the CMOS substrate was etched through [37].

CMOS silicon substrate can also be included in the MEMS structures using a wet etch process. The first method is to perform a time-controlled backside etch with a well-calibrated etching rate. A uniform single crystal silicon membrane with the desired thickness can be created. This method has been widely used in the industry for fabrication of large volume products such as integrated pressure sensors. In cases where the silicon membrane thickness is not critical, even mechanical processing such as grinding can be used to create the backside cavity.

The second method involves the utilization of an automatic etch stop technique to create silicon membranes or MEMS structures. In this case, an anisotropic etch stops at the electrochemically biased PN junction formed between the n-well and p-type substrate in CMOS [38]. Although the electrochemical electrode design and implementation are complicated, this process can be specifically used in the fabrication of highly sensitive pressure/force and thermal sensors. The anisotropic etch stop can also occur at highly doped p- regions in the substrate. This method has been used in fabrication of many suspended structures including neural probes [39]. Note that the p++ doping process may not be available in a standard CMOS process. In the case where only a small portion of the silicon substrate needs to be removed to reduce the circuit-substrate coupling, a wet silicon etch can be performed from the front side. In wet silicon etching, either silicon nitride or additional polymers, or both, can be used to protect the front CMOS and pads.

**Figure 5:**
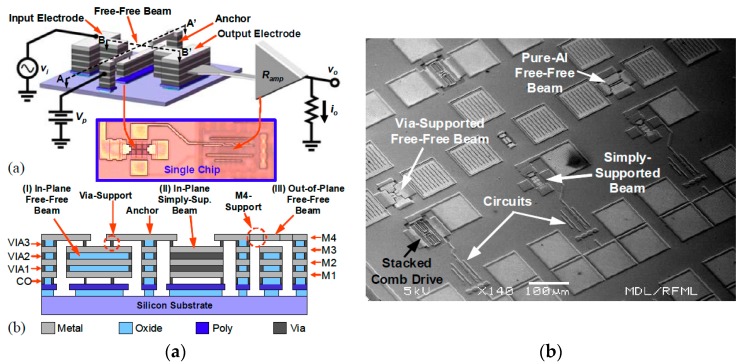
Illustrative structure and circuits of the radio frequency (RF) switch in [40] (**a**), and SEM photograph of the chip (**b**).

CMOS inherent thin films, including multiple metal layers, SiO_2_ insulating layers, and interconnect vias, have been employed as radio frequency (RF) switch and resonator materials. Using wet etching of the thin films, a lot of RF-MEMS components have been produced [40,41]. Figure 5 shows different configurations of capacitively transduced resonators monolithically integrated with their associated amplifier circuits, spanning frequencies from 500 kHz to 14.5 MHz. In formation of the tuning capacitors that are formed by CMOS stacks, SiO_2_ between the microstructures is removed by wet etchant. The circuitry is protected by passivation layers; and the SiO_2_ inside the CMOS beams is protected by the interconnect vias during the device release, *i.e.*, wet removal of SiO_2_. In the integrated absolute pressure sensor in [42], stacked CMOS layers consisting of multiple metal layers, vias and SiO_2_, are used as the movable electrode; the lower metal layer is used as the fixed electrode. An intermediate CMOS metal layer is used as a sacrificial layer and is removed by H_2_SO_4_ + H_2_O_2_ wet etching. CMOS MEMS resonators and accelerometers fabricated using a similar process have also been reported [43,44]. Polymers sensitive to analytes can be coated on finished CMOS MEMS structures for chemical and biological sensing. For example, the first CMOS-MEMS electronic nose was demonstrated by forming polymer-coated CMOS thin film cantilevers on a CMOS chip [45]. In this presentation, vapor hydrofluoric acid based release etching is also categorized as a wet release mechanism. Recently, Baolab Microsystem has commercialized its proprietary NEMS process in an effort to provide a low-cost, high-yield approach for CMOS MEMS integration [46]. In this processing technology, a single vapor hydrofluoric acid (vHF) etching step is used to release the MEMS structure that is consisted of BEOL metal/SiO_2_ stack. The vHF etching step etches away the silicon oxide in between the metal layers of the backend. Depending on the design, the MEMS/NEMS can be built with a combination of stand-alone metal layers and/or stack-ups of metal layers only, and/or metal layers with oxide in between. Following the etching step for MEMS release, an additional Al sputtering and patterning step is available for sealing the cavity, so that any standard package can also be used. An integrated 3D magnetometer has been produced using this integration fabrication module.

Table 2 summarizes some representative devices that were fabricated using wet etching when this technology was dominant in post-CMOS micromachining. Bibliographies of these efforts can be found in the above citations in this section.

**Table 2:** Some CMOS MEMS devices enabled by subtractive process wet etching.

Authors and References	Institutions	Device	Structural Materials	Etching Method	Year
Wise *et al.* [47]	University of Michigan	Pressure sensor	Silicon diaphragm	Backside ethylene diamine-pyrocatechol (EDP) etching	1979
Wise *et al.* [39]	University of Michigan	Neuron probe array	Nitride/SiO_2_, poly and Si substrate	EDP etching, p++ etching stop	1985
Yoon and Wise [48]	University of Michigan	Mass flow sensor	CMOS nitride/SiO_2_, Au/Cr	Backside, SiO_2_ etching stop	1990
Baltes *et al.* [4]	ETH Zurich	Thermal capacitor	Metal/SiO_2_, poly	Front side etching	1996
Haberli *et al.* [49]	ETH Zurich	Pressure sensor	Metal/SiO_2_, poly	Front side etching of aluminum as sacrificial layer	1996
Schneider *et al.* [50]	ETH Zurich	Thermal sensor	Metal/SiO_2_, poly, suspended Si	PN junction electrochemical etch stop	1997
Akiyama *et al.* [51]	University of Neuchatel, ETH Zurich	Atomic force microscope (AFM) probe	CMOS Nitride/SiO_2_, Si	N well electrochemical etch stop	2000
Schaufelbuhl *et al.* [52]	ETH Zurich	Infrared imager	Nitride/SiO_2_, Al, gate poly	Backside KOH	2001
Verd *et al.* [41]	Autonomous University of Barcelona	Integrated Resonator	Al layer	Front side SiO_2_ etching	2006
Chen *et al.* [40]	National Tsing Hua University	Integrated resonator	Al/SiO_2_/Vias	Front side SiO_2_ etching	2011
Narducci *et al.* [42]	IME, Singapore	Absolute pressure sensor	Al/SiO_2_/Vias	Front side Metal and via etching	2013
Li *et al.* [43]	National Tsing Hua University	Integrated Resonator	Al/SiO_2_/Vias	Front side Metal and via etching	2015

#### 5.2.2. Subtractive Post-CMOS MEMS by Dry Etching

Plasma enhanced dry etching processes have quickly become prevalent in microfabrication for both MEMS research and industry. Particularly, the deep reactive ion etching (DRIE) technology, or Bosch process, patented by Robert Bosch GmbH, has revolutionized subtractive post-CMOS microfabrication [53]. This section describes thin-film and bulk CMOS MEMS devices fabricated using a dry etching processes.

Most dry etching processes are based on plasma processes, such as reactive-ion etch (RIE) and DRIE. An etching process employing etchants in vapor phase can also be considered a “dry” one. For example, vapor XeF_2_ provides good isotropic etching of silicon, which has been used for releasing CMOS thin film MEMS structures [54]. The combination of RIE and DRIE, performed from the front or back side, or both sides, has allowed for fabrication of a large spectrum of CMOS-MEMS devices. Depending on the structural materials and etching methods employed, subtractive post-CMOS can be divided into two types: thin-film processes and bulk processes.

##### Thin-Film Post-CMOS MEMS Dry Processes

**Figure 6:**
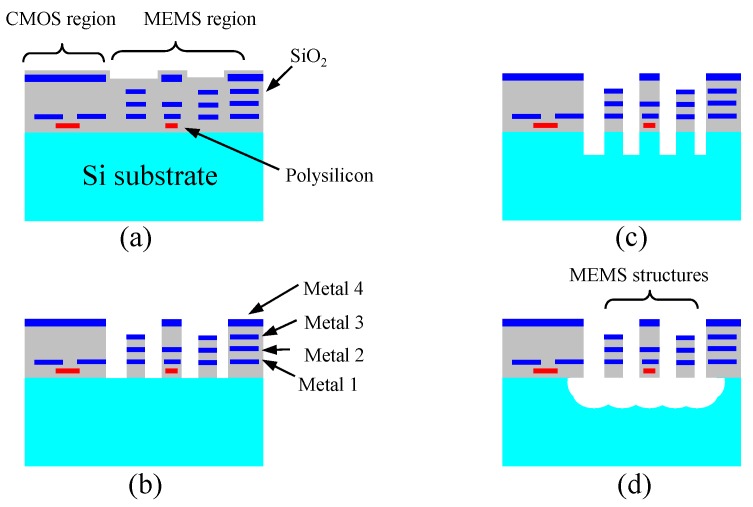
Dry-etching-based post-CMOS fabrication process for MEMS structures made of CMOS thin films [57]. (**a**) CMOS wafer or die; (**b**) SiO_2_ etching; (**c**) Silicon deep reactive ion etching (DRIE); (**d**) Silicon reactive ion etching (RIE) with lateral undercut.

**Figure 7:**
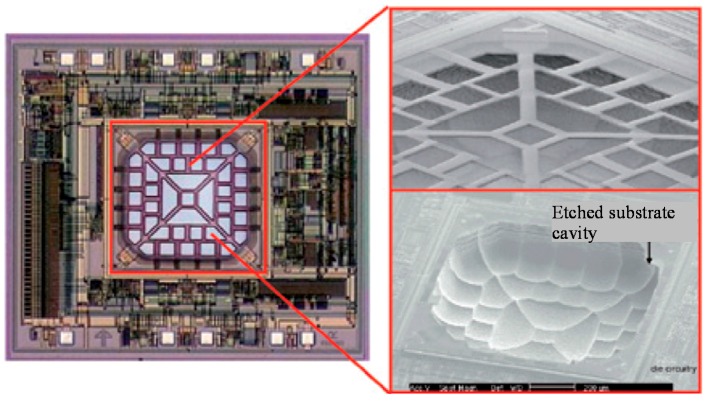
Die photo and SEM image of an integrated convective accelerometer from MEMSIC [68].

In thin-film processes, structural materials are composed of inherent CMOS thin films. A very wide spectrum of MEMS devices developed and commercialized recently falls into this category. In this review, the post-CMOS MEMS process originally developed at Carnegie Mellon University (CMU) is the main focus. Figure 6 depicts a characteristic process flow of CMU’s post-CMOS thin film process [55]. Sequenced processes consisting of an isotropic SiO_2_ etching, a silicon DRIE and an isotropic Si RIE undercut expose, define and release the MEMS structure, respectively. In these process steps, the top metal layer acts as a mask to form the MEMS structures and to protect the CMOS circuitry, as seen in Figure 6a,b. Anisotropic and isotropic silicon etching complete the process flow, as seen in Figure 6c,d. Various inertial sensors have been fabricated using this thin film technology [56,57,58]. In all these inertial sensors, mechanical springs and proof masses are formed by the multiple-layer CMOS stacks consisting of SiO_2_ and metals. The sensing capacitance is formed from sidewall capacitance between comb fingers. The multiple CMOS metal layers inside the comb fingers and other mechanical structures allow very flexible electrical wiring, facilitating different sensing schemes including vertical comb-drive sensing. Variations of the sensing structures have been used for other sensors and actuators [59,60,61,62,63,64]. Using its proprietary copper “Damascene” technology in which both polysilicon and polymers are used as sacrificial layers and removed by RIE etching, IBM has produced a number of RF MEMS passive components with stacked CMOS layers in its 0.18 μm copper CMOS technology [65,66]. In industry products, Akustica Inc., now a subsidiary of Bosch GmbH (Gerlingen, Germany), has commercialized digital microphones using a modified version of the process in Figure 6 [67]. MEMSIC (Andover, MA, USA), an inertial sensors provider, has utilized CMOS-MEMS stacks and silicon RIE in manufacturing its series convective MEMS accelerometer. Figure 7 shows a photograph and SEM image of a die of convective accelerometer from MEMSIC [68].

Despite the excellent CMOS compatibility, flexible foundry accessibility of the above thin film post-CMOS dry etching processes, a major issue exists. For some structures where dimension variations are critical, the large vertical curling and lateral buckling of the suspended MEMS structures that are caused by the residual stress in the stacked thin-film CMOS layers pose a challenge for device performance. Although structural curling can be tolerated for some small devices such as RF MEMS and thermal sensors, for devices such as inertial sensors that need relatively large size, the impact of structural curling can be severe, and compensation may be mandatory [69]. Moreover, the need for etching access holes in fabrication limits the size and mass of the devices.

##### Bulk CMOS-MEMS Dry Process

**Figure 8:**
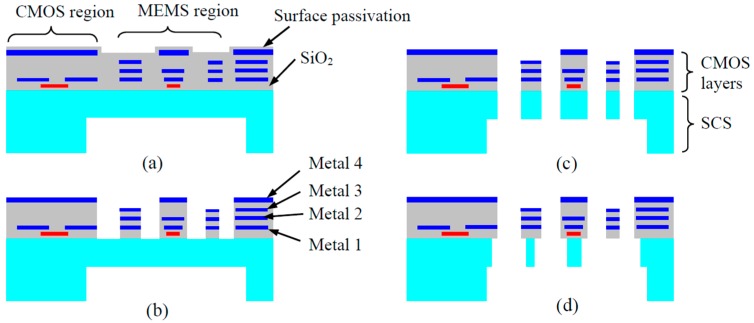
DRIE bulk CMOS-MEMS process flow for 4-metal-layer CMOS [70]. (**a**) Backside silicon DRIE to define MEMS areas; (**b**) Front SiO_2_ etching; (**c**) Front-side silicon DRIE; (**d**) Front-side RIE with lateral undercut.

In order to overcome the structural curling and to increase the mass, flatness and robustness of MEMS structures, single crystal silicon (SCS) may be included underneath the CMOS thin-film stacks. The SCS silicon structures are formed directly from the silicon substrate using DRIE. Figure 8 illustrates the process flow, in which a 4-metal-layer CMOS technology is used as an example [70]. The process starts with the backside silicon DRIE to define the MEMS structure thickness by leaving a 10–100 μm-thick SCS membrane (Figure 8a). Next, the same anisotropic SiO_2_ etch as in the thin film process is performed on the front side of wafer (chip) to expose the SCS to be removed (Figure 8b). The following step differs from the thin film process in that an anisotropic DRIE, instead of isotropic etch, finalizes the structure release by etching through the remaining SCS diaphragm, as shown in Figure 8c. With the SCS underneath the CMOS interconnect layers included, large and flat MEMS microstructures can be obtained. If necessary, an optional time-controlled isotropic silicon etch can be added. This step will undercut the SCS underneath the designed narrow CMOS stacks to create thin film structures (Figure 8d). This step is particularly useful in fabrication of capacitive inertial sensors. It can be used to form the electrical isolation structures between sensing electrodes and silicon substrate.

**Figure 9:**
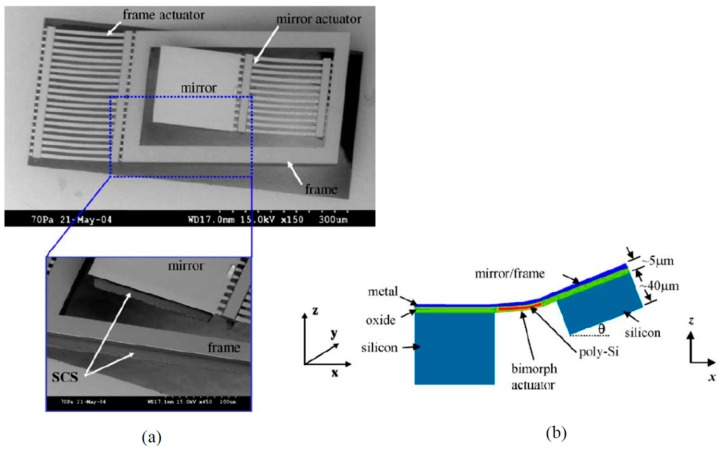
SEM images and structure illustration of an electro-thermal micromirror by bulk post-CMOS microfabrication [71]. (**a**) Micrographs of the mirror and microstructures; (**b**) Illustrative composition of the mirror structures.

The DRIE CMOS-MEMS technology has shown great advantages in the fabrication of relatively large MEMS devices such as micromirrors [71]. Large flat mirror can be obtained by including portion of silicon substrate underneath the aluminum mirror surface, as shown in Figure 9. A CMOS-MEMS gyroscope with a low noise floor permitted by the SCS proof mass has also been produced using this technology [72]. More recently, a couple of *z*-axis accelerometers with large proof mass have been fabricated using this technology [73,74].

By attaching SCS underneath the CMOS stack comb fingers, the sensing capacitance of capacitive sensors can be considerably increased for a higher signal-to-noise ratio (SNR). Although CMOS thin films are still used in some microstructures for electrical isolation, the length of the thin-film portion is minimal to reduce the temperature effect. Compared to the thin film dry CMOS-MEMS process, a backside silicon DRIE step is added to define the thickness of the silicon to be included. This requires an additional backside lithography step to define the region for MEMS structures. The maximum thickness of the MEMS structures is limited by the aspect ratio that the silicon DRIE can achieve.

##### An Improved Bulk CMOS MEMS Process

The bulk CMOS MEMS process depicted in Figure 8 is useful in fabrication of many devices where SCS structures are desired to improve both mechanical and electrical performance of the devices. However, for some devices, very fine structures are formed in step (c) in Figure 8, so the damage caused by the step (d) to these fine structures may be severe. This is particularly true for the fabrication of capacitive inertial sensors where narrow-gap sensing comb fingers are needed. For instance, in performing the isotropic silicon undercut to form the narrow CMOS beams for electrical isolation and mechanical connection, the SCS in the comb fingers is also undercut. The sensing gap increases due to the undesired undercut, and consequently, the sensing capacitance reduces sensitivity and signal-to-noise ratio (SNR) of the sensor degrade. If the undercut occurs in mechanical structures such as suspension springs, the dynamic characteristics of the device will also be severely affected. Another issue is related to the thermal effect in the plasma etch for the SCS undercut. Upon completion of the silicon undercut, the greatly reduced thermal conductance from the isolated structure to the substrate can cause a temperature rise on the released structures. Slight over-etch is often necessary to accommodate process variations, but this will generate a large temperature increase in the suspended structures which in turn dramatically increases the SCS etching rate, resulting in uncontrollable and damaging results.

**Figure 10:**
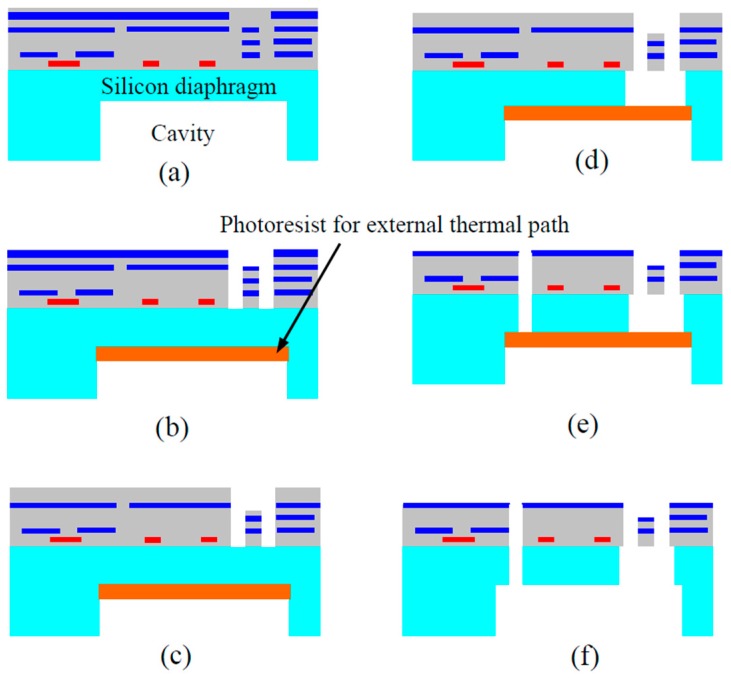
Modified bulk CMOS MEMS process for separate etching of CMOS beams and single crystal silicon (SCS) microstructures [75]. (**a**) Backside silicon DRIE; (**b**) Thermal protection using photoresist (PR); (**c**) Front-side SiO_2_ etching; (**d**) Front-side silicon DRIE for beams; (**e**) Front-side silicon DRIE for comb drives; (**f**) Removal of thermal protection PR layer.

**Figure 11:**
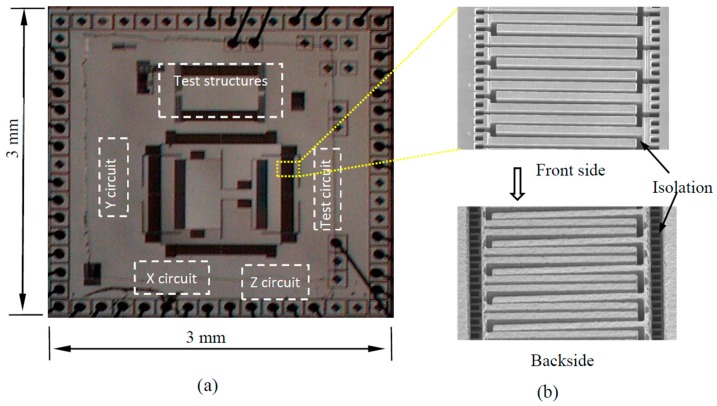
Die photo (**a**) and SEM micrograph of microstructures in the sensor (**b**) reported in [76].

A modified dry bulk CMOS MEMS process has been demonstrated to effectively address the issues caused by the undesired SCS undercut [75]. In the refined process illustrated in Figure 10, the etching of the CMOS isolation/connection beams is performed separately from the etching of the microstructures where SCS is needed. The top metal layer is purposely used to define the isolation/connection beams. After their formation, the top metal layer is removed using a plasma or a wet etch. Then other microstructures are exposed after a SiO_2_ etch. The direct etch-through of the remaining silicon on the microstructures will complete the release process. To reduce the thermal effect described above, a thick photoresist layer is patterned on the backside of the cavity. In the release step, the applied photoresist provides a thermal path that reduces the temperature rise on the etched-through structures. The removal of the photoresist using O_2_ plasma etching completes the entire microfabrication process. Owing to the monolithic integration and large proof mass enabled by the inclusion of SCS, bulk CMOS MEMS inertial sensors have demonstrated better performance than their thin-film counterparts [76]. Figure 11 shows a 3-axis accelerometer fabricated using the improved bulk CMOS MEMS process that is pictured in Figure 10.

Recently, to address the etching deterioration caused by the thermal effect first reported in [75], some new approaches, including multiple step etching [77,78], and backside coating of a metal layer to provide thermal dissipation path [79,80]. A CMOS MEMS accelerometer, gyroscope and micromirror with large mirror plate have been fabricated using the further improved methods, respectively.

#### 5.2.3. Combined Wet/Dry Processes

In addition to the integration methods described above, efforts have been continuously made to integrate CMOS with MEMS using the combination of different microfabrication technologies. By combing silicon anisotropic wet etch with DRIE, some sophisticated surface and bulk MEMS structures such as bridges and cantilever arrays can be created. A multi-sensor system was produced using a combined etch process [81]. In the accelerometers reported in [82], isotropic wet etching is used to remove metal layers in CMOS thin stacks to create parallel plate-like vertical capacitors for gap-closing sensing. A silicon RIE follows to release the MEMS devices and break the coupling between the sensing thin films and the substrate. Compared to conventional in-plane comb-finger sensing, the sensor features improved sensitivity that is attributed to vertical gap-closing sensing enabled by the new fabrication approach. In another effort, combination of SiO_2_ wet etching and XeF_2_ isotropic silicon etch is responsible for implementation of symmetric structures leading to accelerometer performance improvement [83]. In the absolute pressure sensor reported in [84], a micro vacuum chamber is created by sealing the etching access holes using a LPCVD parylene thin film upon completion of wet metal etching for sensing diaphragm formation. The parylene film is then patterned and removed by dry etching.

A high Q CMOS MEMS variable capacitor for RF application has been implemented by combination of multiple dry and etching steps [85]. Stacked CMOS layers of a commercial 0.35 μm technology were used as electrode material. A total of 3 dry etching steps and 4 wet etching process were conducted in SiO_2_, metal and silicon substrate etching, respectively.

In demonstration of the integrated gyroscope in [78], the authors employed TMAH backside anisotropic silicon etching to mitigate the undercut on SCS in the comb drives resulting from the footing effect in front side DRIE. A SiO_2_ layer by wet oxidation was coated in the trenches formed by front DRIE etching. It serves as protection and etch stop layer during the backside SCS wet etch. The MEMS structure was released after the SiO_2_ was removed by buffered oxide etch (BOE).

Using combined dry/wet etching approaches, integration of structures with nanometer dimensions with conventional CMOS structures have also been vigorously explored. An excellent recent review on relevant devices by Uranga *et al.* has summarized the efforts in this exciting area [21]. In [86,87], arrays of single crystal silicon oscillators integrated with closed-loop control scheme have been fabricated. The implementation of such arrays not only improves the sensitivities of oscillator elements thus overall detection efficiency, but also paves the way for new applications of integrated NEMS that require large detection cross section and individual addressing, such as in spectroscopy.

### 5.3. Silicon-on-Insulators (SOI) CMOS MEMS Technologies and Devices

Enabled by smart-cut and other technologies, silicon-on-insulator (SOI) wafers have been made largely commercially available for CMOS and MEMS community. In addition to its great advantages in manufacturing high performance CMOS circuits thanks to its excellent electrical isolation, SOI has been demonstrated as an excellent option for MEMS fabrication as well. SOI-CMOS MEMS integration has been aggressively explored ever since the material became available [88,89,90,91]. In SOI MEMS, the top single crystal silicon layer with thickness ranging from a few microns to tens of micrometers has been adopted. When used as a structural layer, compared with those in the bulk CMOS MEMS structures, the SOI MEMS has a better dimensional control in DRIE-based structure release. Despite some physical effects involved in the DRIE process of the SOI layer, such as footing effect that slightly undercuts the bottom of the SOI trench at the buried SiO_2_ layer, the thickness of the SOI MEMS can normally be retained as its well-defined thickness in material preparation [92]. Some special designs have been demonstrated to compensate for the footing effect in SOI MEMS processes [93]. Overall, the process for MEMS portion of SOI CMOS MEMS is advantageous over the widely used double side DRIE processes in which less accurate timing control is responsible for structure thickness determination, as described earlier. Therefore, SOI CMOS MEMS provides a unique approach in integration of high performance CMOS or BiCMOS circuitry with single crystal silicon MEMS structure, featuring good dimensional control. Representative products include Analog Devices’ ADXL001 (Analog Devices Inc., Norwood, MA, USA) integrated accelerometer and ADXRS453 integrated gyroscope (Analog Devices Inc.) which can be operated at a high temperature of 130 °C [94].

In SOI CMOS MEMS, interconnects between MEMS and CMOS portion should be carefully arranged. In Analog Devices’ SOI CMOS MEMS accelerometers [17], polysilicon plugs are employed for interconnection and isolation between movable MEMS structure and CMOS circuitry. In [95], a “micro bridge” on which additional aluminum was patterned serves as connection between the CMOS and MEMS. The demonstrated accelerometers were fabricated using an in-house SOI CMOS process. A unique below IC integration of SOI MEMS has been employed with an NXP/Philips semiconductor (NPX Semiconductors, Eindhoven, The Netherlands) as an approach to fabricate various devices [96]. In contrast to most methods in which top silicon layer is used for MEMS structure, in this approach, bulk substrate is thinned down as thick MEMS structure and is connected to CMOS circuits located in thin top Si layer through polysilicon plug in thin SOI layer.

In addition to the devices described in the above technological literatures, a large variety of other SOI CMOS MEMS devices have been demonstrated. Authors in reference [97] reported a MEMS micro hotplate enabled by tungsten film with SOI CMOS technology. The hotplate can be used as a platform for design of some gas sensors. Imaging sensors and micromirror arrays have also been reported [98,99,100].

With new integration technologies such as silicon vias (TSV) being matured, SOI CMOS MEMS will play a significant role in implementation of system-on-chip (SOC) and other complex microsystems [101,102].

For the research community, in choosing a CMOS-MEMS monolithic integration approach, the following general considerations should be followed: (1) Performance requirements of the designed device and system. For instance, for inertial sensors, bulk CMOS-MEMS and SOI CMOS-MEMS can provide proof mass normally thicker than thin-film counterparts for improved resolutions. However, the performance balance between the CMOS circuitry and MEMS devices should be highly sought after. (2) Availability of foundry services and critical design rule requirements from particular CMOS foundries. In many of the CMOS MEMS devices fabricated using the process derived from the CMU approach, a large array of vias is placed as vertical electrodes when vertical capacitors are needed. Due to the CMOS design rules on the vias, the electrodes are actually non-continuous. (3) Intrinsic effects of CMOS materials on MEMS structures. Since CMOS technologies and materials by nature are not optimized for MEMS devices, physical effects such as residual stress, material texture, discrepancies in thermal expansion coefficients, *etc.*, should be well elaborated in MEMS design and fabrication. More and more CMOS MEMS foundries are starting to provide general guidance and process data for MEMS design, yet consistency of post CMOS-fabrication results still need improvement. (4) Fabrication capability of in-house facility. When post CMOS fabrication is performed in-house, equipment capability should be well calibrated and associated data should be used as part of MEMS design rules. (5) Balance between cost and device performance. Although most research projects are technology- and performance-driven, cost, process scalability should also be considered, especially when there is a possibility of commercialization of the technology and system under development.

## 6. Summary and Future Trends for System-On-Chip (SOC)

CMOS-MEMS technologies have been placed in pre-CMOS, intra-CMOS and post-CMOS categories. Both pre-CMOS and intra-CMOS have issues such as dedicated foundries with suboptimal and less cost-effective processes. Post-CMOS provides excellent CMOS compatibility, foundry accessibility and design flexibility, and the overall cost is reduced. SOI-CMOS MEMS have also been aggressively explored. The technologies involved in this new integration approach, such as TSV and wafer bonding, have allowed 3D integration and wafer level packaging of CMOS circuitry and MEMS, to form complex microsystems such as system-on-a-chip (SOC), a term borrowed from stacked ICs [103,104]. The new integrations has blurred the boundary between pre- and post-CMOS MEMS integrations.

In MEMS industry, on one hand, individual fabrication processes and integrations have been tremendously improved by enabling technological breakthroughs and equipment. For instance, major MEMS suppliers, including STMicroelectronics and InvenSense, have adopted wafer-to-wafer or chip-to-wafer bonding CMOS MEMS integration. On the other hand, technological trends have been shifted to creation of common platforms for both CMOS and MEMS. A number of CMOS foundries, including Taiwan Semiconductor Manufacturing Company, X-Fab and Global Foundries, *etc.*, have begun to offer CMOS MEMS services for research and product development. According to the international roadmap for the semiconductor industry, one of the current challenges of CMOS-MEMS integration technology is the modification and standardization of CMOS technology to accommodate MEMS technology [105]. It can be anticipated that another wave of innovations in CMOS MEMS integration will quickly push the microelectronic industry to a new era of “more than Moore”.
